# A novel experimental rat model of peripheral nerve scarring that reliably mimics post-surgical complications and recurring adhesions

**DOI:** 10.1242/dmm.028852

**Published:** 2017-08-01

**Authors:** Angela Lemke, Carina Penzenstadler, James Ferguson, Dominika Lidinsky, Rudolf Hopf, Monika Bradl, Heinz Redl, Susanne Wolbank, Thomas Hausner

**Affiliations:** 1 Ludwig Boltzmann Institute for Experimental and Clinical Traumatology/AUVA Research Center, Donaueschingenstraße 13, Vienna 1200, Austria; 2 Austrian Cluster for Tissue Regeneration, Austria; 3 Department for Neuroimmunology, Center for Brain Research, Medical University Vienna, Spitalgasse 4, Vienna 1090, Austria; 4 Department of Traumatology, Lorenz Böhler Hospital, Donaueschingenstraße 13, Vienna 1200, Austria; 5 Department for Trauma Surgery and Sports Traumatology, Paracelsus Medical University, Strubergasse 21, Salzburg 5020, Austria

**Keywords:** Peripheral nerve adhesions, Perineural adhesions, Nerve fibrosis, Nerve scarring, Nerve inflammation

## Abstract

Inflammation, fibrosis and perineural adhesions with the surrounding tissue are common pathological processes following nerve injury and surgical interventions on peripheral nerves in human patients. These features can reoccur following external neurolysis, currently the most common surgical treatment for peripheral nerve scarring, thus leading to renewed nerve function impairment and chronic pain. To enable a successful evaluation of new therapeutic approaches, it is crucial to use a reproducible animal model that mimics the main clinical symptoms occurring in human patients. However, a clinically relevant model combining both histological and functional alterations has not been published to date. We therefore developed a reliable rat model that exhibits the essential pathological processes of peripheral nerve scarring. In our study, we present a novel method for the induction of nerve scarring by applying glutaraldehyde-containing glue that is known to cause nerve injury in humans. After a 3-week contact period with the sciatic nerve in female Sprague Dawley rats, we could demonstrate severe intra- and perineural scarring that resulted in grade 3 adhesions and major impairments in the electrophysiological peak amplitude compared with sham control (*P*=0.0478). Immunohistochemical analysis of the nerve structure revealed vigorous nerve inflammation and recruitment of T cells and macrophages. Also, distinct nerve degeneration was determined by immunostaining. These pathological alterations were further reflected in significant functional deficiencies, as determined by the analysis of relevant gait parameters as well as the quantification of the sciatic functional index starting at week 1 post-operation (*P*<0.01). Moreover, with this model we could, for the first time, demonstrate not only the primary formation, but also the recurrence, of severe adhesions 1 week after glue removal, imitating a major clinical challenge. As a comparison, we tested a published model for generating perineural fibrotic adhesions, which did not result in significant pathological changes. Taken together, we established an easily reproducible and reliable rat model for peripheral nerve scarring that allows for the effective testing of new therapeutic strategies.

## Abstract (precis)

**Summary:** An easily reproducible and reliable rat model for peripheral nerve scarring that allows for the effective testing of new therapeutic strategies.

## INTRODUCTION

Scarring and adhesion represent major challenges following injury and surgical procedures of peripheral nerves, often leading to pain and even nerve dysfunction. Currently, the removal of the surrounding scar tissue (external neurolysis) up to the resection of the epifascicular fraction of the epineurium (internal neurolysis) is the most common treatment (Mazal and Millesi, 2005; Okui et al., 2010; Frykman et al., 1981; Sakurai and Miyasaka, 1986; Calfee et al., 2008). However, clinical symptoms recur in most patients owing to re-appearing secondary perineural adhesions and neural fibrosis (Abe et al., 2005; Botte et al., 1996; Dam-Hieu et al., 2005; Frykman et al., 1981; Ohsumi et al., 2005; Robertson, 1996; Rydevik et al., 1976), resulting in impaired nerve function (Lundborg, 2003; Ngeow, 2010; Ohsumi et al., 2005). Therefore, several different therapeutic approaches have been tested (Doi et al., 1992; Varitimidis et al., 2001; Wintsch and Helaly, 1986), including implantation of muscle flaps, fat grafts (De Smet and Vandeputte, 2002; Bernsmann et al., 2001) or diverse biomaterials (Dam-Hieu et al., 2005; Ikeda et al., 2003; Ohsumi et al., 2005; Petersen et al., 1996; Wang et al., 1998), yet most therapies are still experimental and none are routinely used on patients (Atkins et al., 2006; Ngeow, 2010; Okui et al., 2010).

For the successful evaluation of the efficacy of new treatments against fibrotic adhesions of peripheral nerves, it is essential to have a reliable and reproducible animal model that mimics the common clinical characteristics. Also, it should meet objectively quantifiable parameters to measure therapeutic success and function of the corresponding tissue (Muschler et al., 2010; Zanjani et al., 2013). In general, there are two different methods of inducing neural scar tissue formation: either by inducing a direct lesion by applying physical (Crosio et al., 2014; Dam-Hieu et al., 2005; Görgülü et al., 2003; Özay et al., 2007; Smit et al., 2004; Zuijdendorp et al., 2008), chemical (Wachter et al., 2002) or thermal force (Abe et al., 2005; Ikeda et al., 2003), or by initiating indirect damage to the nerve surface through injury of the surrounding muscular bed (Albayrak et al., 2010; Crosio et al., 2014; Dam-Hieu et al., 2005; Ilbay et al., 2005; Palatinsky et al., 1997; Zanjani et al., 2013). However, an ideal, easily reproducible and authentic model combining morphological, electrophysiological and especially functional outcomes in which the main consequences of peripheral neuropathy can be simulated was not available (Zanjani et al., 2013). The majority of published animal models can reliably demonstrate perineural adhesions by gross and histological evaluation but not functional deficiencies, such as gait pattern impairment (Abe et al., 2005; Crosio et al., 2014; Dam-Hieu et al., 2005; Ilbay et al., 2005; Petersen et al., 1996; Smit et al., 2004; Zanjani et al., 2013). Moreover, these studies usually do not investigate the recurrence of adhesions in these models, which is a major complication in human patients.

In this study, we addressed these issues by establishing a novel rat model for the chemical induction of intraneural fibrosis and perineural adhesions by combining direct and indirect approaches of generating neural damage and perineural *de novo* adhesions. To assess the efficacy of our new model, we compared it with an already published scratch model (Crosio et al., 2014; Görgülü et al., 2003; Petersen et al., 1996), in which adhesions with the surrounding tissue are caused by irritating the nerve surface with a cotton swab. By exposing the sciatic nerve to different amounts of a mixture of serum albumin and glutaraldehyde for a 3-week period, we were able to reliably evoke nerve inflammation, fibrosis and perineural adhesions, simulating the major processes of peripheral neuropathy. Moreover, we could demonstrate the spontaneous occurrence of severe secondary adhesions after external neurolysis, as is common in human patients.

## RESULTS

### Morphological examination of *de novo*-formed perineural fibrotic adhesions

**Fig. 1.**
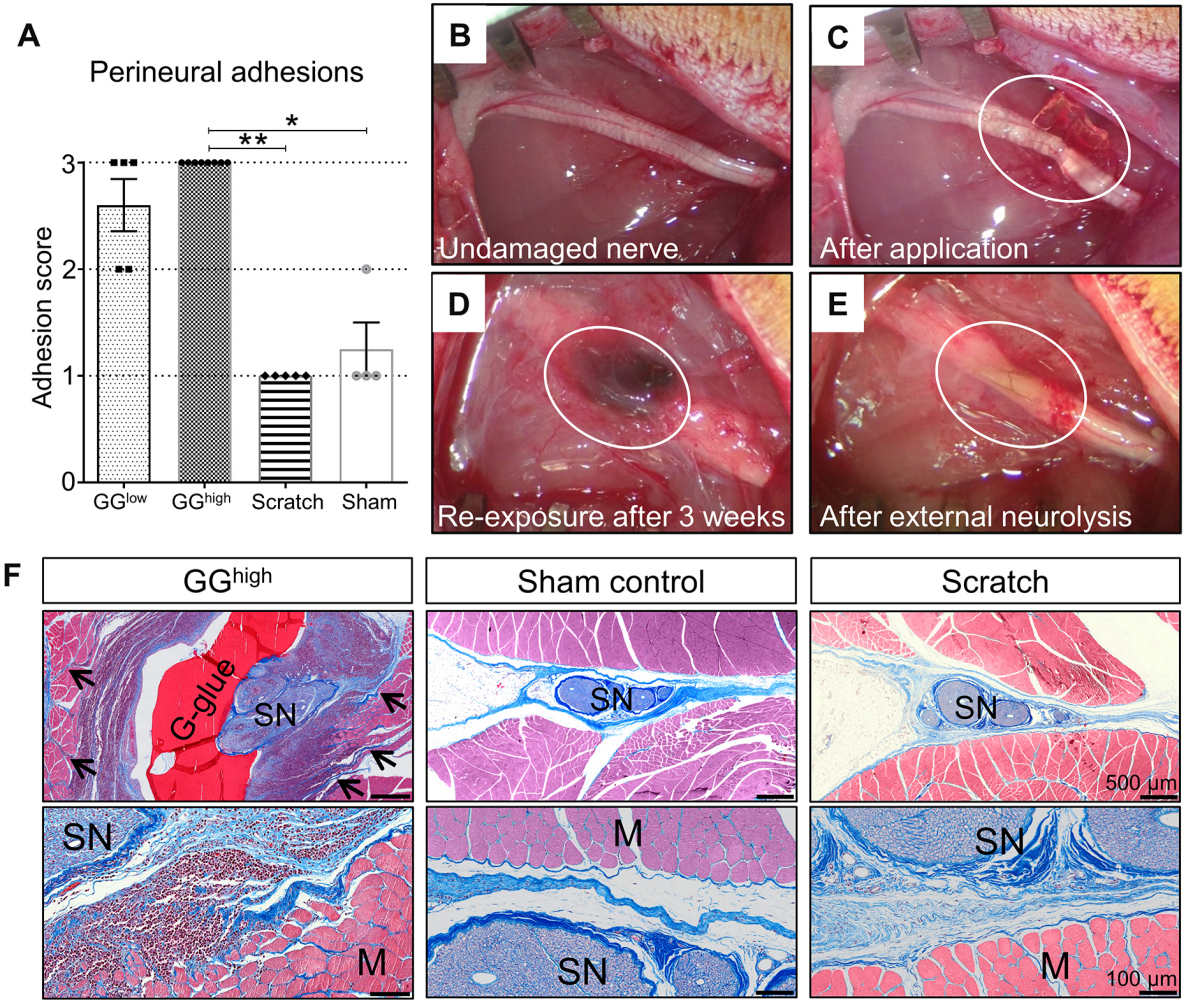
**Analysis of perineural adhesions between the sciatic nerve and the surrounding tissue 3 weeks after primary surgery.** (A) Rats belonging to the GG^high^ group (*n*=8) exhibited distinct adhesive fibrotic tissue requiring sharp dissection during external neurolysis, whereas sham (*n*=4, *P*=0.0125) and scratch (*n*=5, *P*=0.0012) groups had no or mild adherence . Rats of the GG^low^ group (*n*=5) also showed predominantly severe adhesions. The difference between the GG^low^ group and the other groups was, however, not statistically significant (*P*=0.2129 and *P*=0.0562). *P<0.05, **P<0.01. (B-E) Morphological gross evaluation of the sciatic nerve. Circled area shows the sciatic nerve immediately after glue application (C), 3 weeks afterwards during re-exposure (D) and after removal of the glue and the developed adhesions (E; external neurolysis). (F) Histological en bloc cross sections of GG^high^, sham control and scratch groups stained with CAB, showing the sciatic nerves and surrounding tissue 3 weeks following primary surgery. The application of glutaraldehyde glue induced strong inflammatory mononuclear cell infiltration of both nerve and muscles, as well as severe growth of dense collagenized matrix infiltrating muscle fibres (arrows). Although, in the scratch group, a slight increase of loose connective tissue could be observed, the surrounding muscles were not affected, similar to sham controls, which showed only mild formation of collagen. G-glue, glutaraldehyde glue; SN, sciatic nerve; M, muscle fibres.

Re-exposure of the sciatic nerves 3 weeks after primary surgery revealed no obvious fibrosis formation in the sham-operated control group (nerve-exposure only), as expected. Except for one rat, which had developed a few fibrotic tissue fibers, all rats were assigned grade 1 of the adhesion score (1.25±0.5, Fig. 1A) in a blinded evaluation, as defined in Table 1. Similar results were obtained for the scratch group (mechanical irritation with cotton swab), which did not reveal any considerable growth of connective tissue (1.00±0, Fig. 1A). In contrast, the 3-week contact period with glutaraldehyde glue (Fig. 1B-E) induced the formation of severe perineural adhesions with the surrounding tissue, requiring sharp dissection in all (at least 50 µl of glutaraldehyde glue: ‘GG^high^’, 3±0) or most (two drops of not more than 10 µl each of glutaraldehyde glue: ‘GG^low^’, 2.60±0.55, Fig. 1A) cases. Large amounts of connective tissue had formed surrounding the glue and the nerve segment (Fig. 1D). Moreover, in the GG^high^ group, external neurolysis revealed intense damage to the epineurium, nerve swelling and increased vascularization (Fig. 1E). The findings among the different experimental groups were reconfirmed by analyzing en bloc cross sections stained with chromotrop aniline blue (CAB) (Fig. 1F), which additionally showed dense collagen fibres invading the muscle bed and high amounts of mononuclear inflammatory cells in the GG^high^ group. On the other hand, a reduced exposure time to glutaraldehyde glue failed to induce distinct perineural adhesion with the surrounding tissue (Fig. S1). Interestingly, a 1-week contact with the glue was, however, already sufficient to induce extensive neural damage (Fig. S2).

**Table 1.**
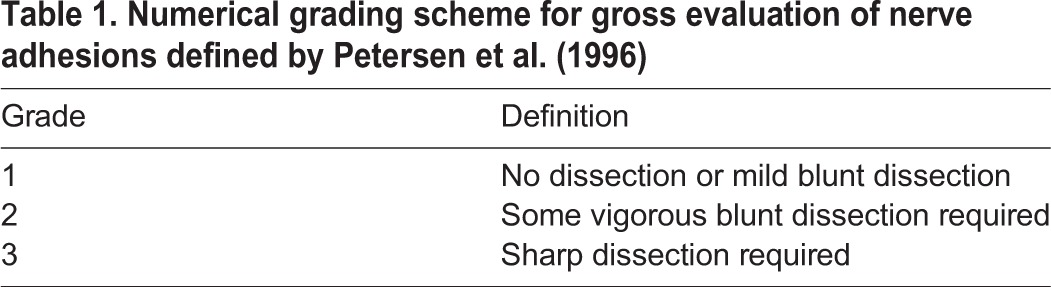
**Numerical grading scheme for gross evaluation of nerve adhesions defined by** **Petersen et al. (1996****)**

### Analysis of electrophysiological signal conduction

**Fig. 2.**
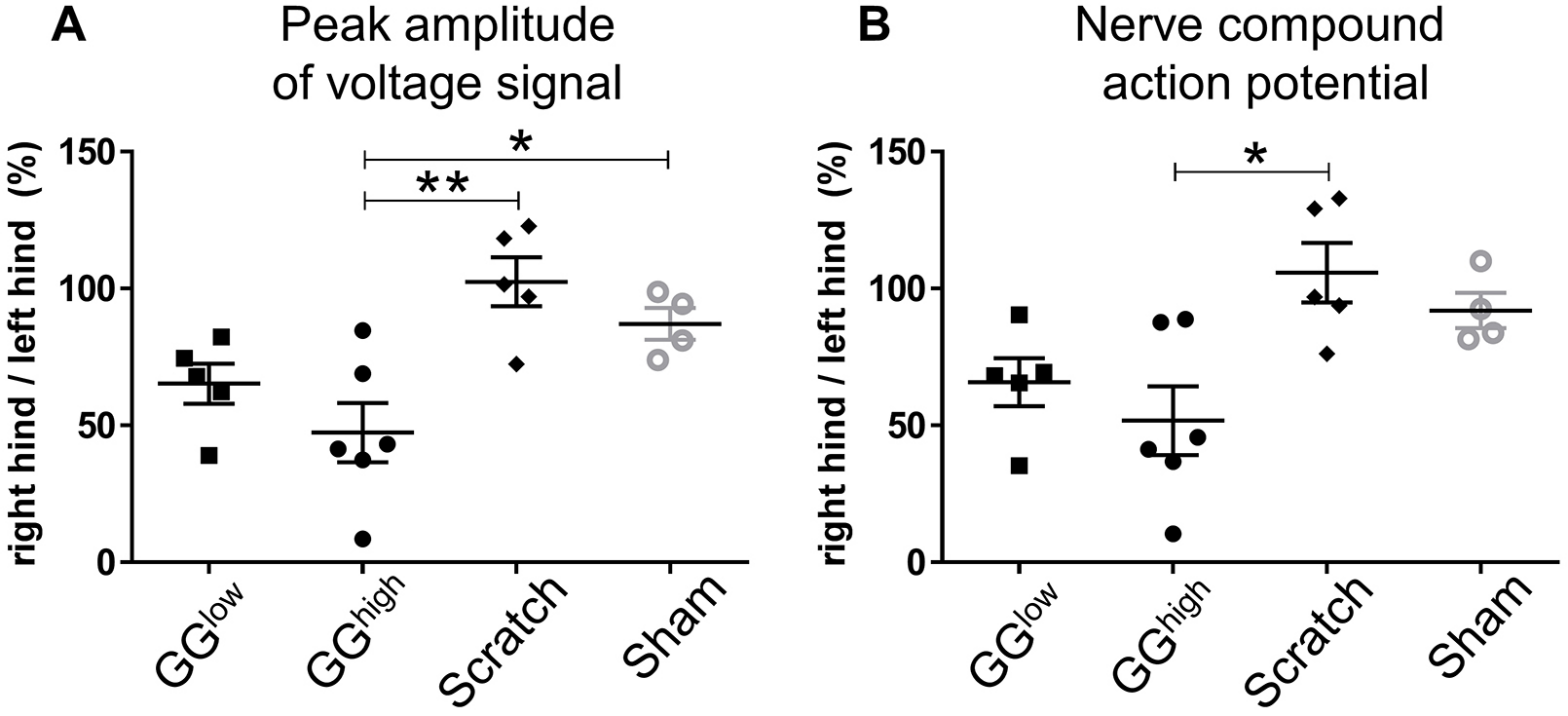
**Peak amplitude of voltage signal and nerve compound action potential in experimental groups at 3 weeks post-operation.** The data for peak amplitude (A) and nerve compound action potential (B) are presented as the ratio of the right hind limb signal to the left hind limb signal, shown as a percentage (×100). GG^low^: *n*=5, GG^high^: *n*=6, scratch: *n*=5, sham-operated control: *n*=4. *P<0.05; **P<0.01.

As shown in Fig. 2A, the peak amplitude of the voltage signal (presented as the ratio of the right hind limb signal to the left hind limb signal) was significantly decreased in the GG^high^ group (47.4±26.5%) 3 weeks after surgery, in comparison to scratch (102.4±20.0%, *P*=0.0023) and sham control (87.1±11.6%, *P*=0.0478). Also, the peak amplitude of the GG^low^ group (65.2±16.4%) revealed minor signal impairment compared to sham (*P*=0.7694). Nerve compound action potential (NCAP) was then analyzed between the experimental groups (Fig. 2B). Although a clear reduction in the GG^high^ group (51.8±30.8%) and a smaller decline in the GG^low^ group (65.8±19.7%) was determined, the difference was only statistically significant between GG^high^ and scratch (105.8±24.4%, *P*=0.0111), but not compared to sham control (92.0±12.9%, *P*=0.1160).

### Histopathological evaluation of intraneural degeneration and fibrosis

**Fig. 3.**
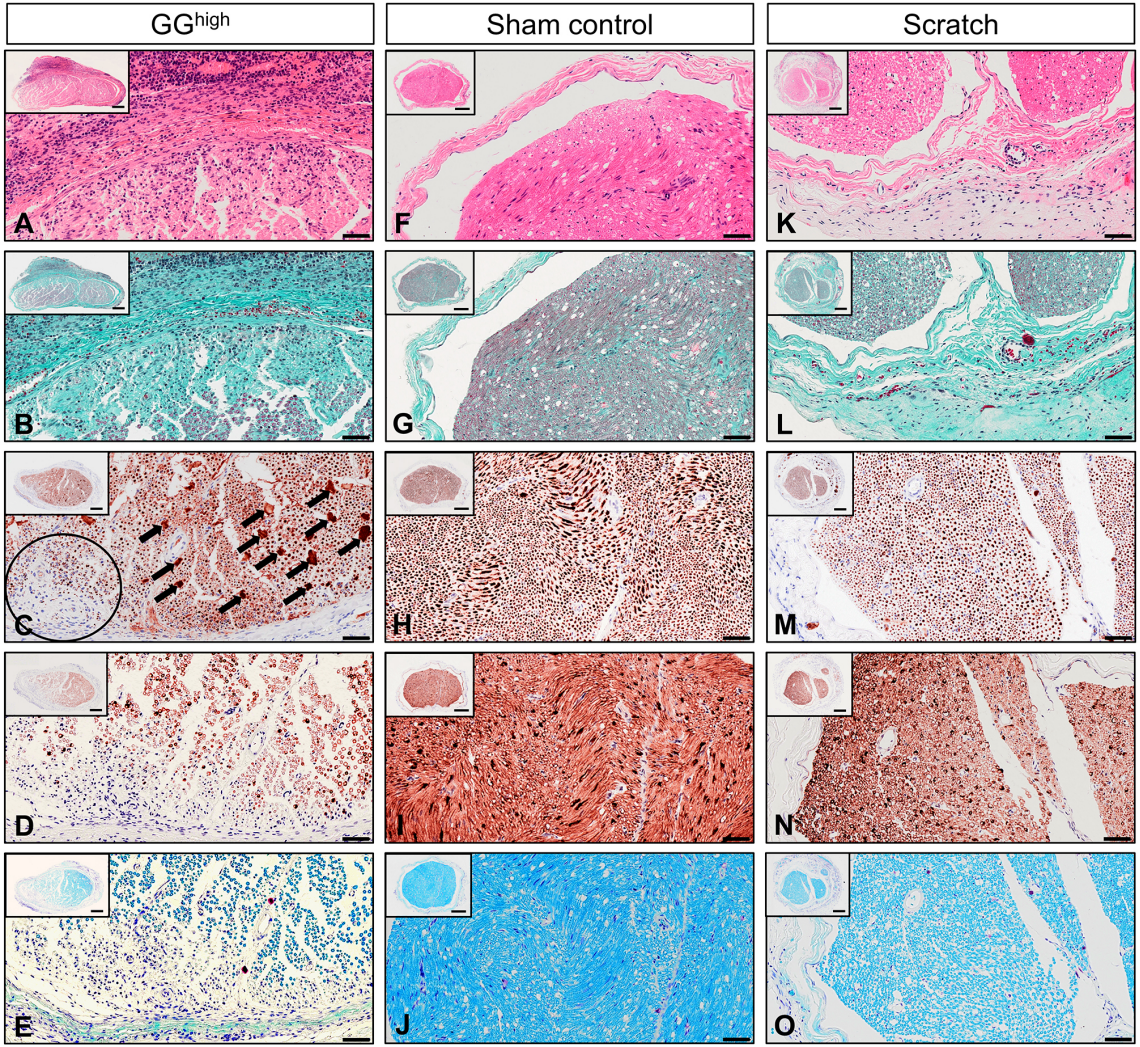
**Representative photomicrographs showing cross sections of the right sciatic nerve of GG^high^, sham control and scratch group at 3 weeks after primary surgery.** Sections were stained with (A,F,K) H&E, (B,G,L) Masson's trichrome, (C,H,M) anti-neurofilament protein, (D,I,N) anti-S100 and (E,J,O) Luxol fast blue. The nerves treated with GG^high^ exhibited (A) severe inflammatory cell infiltration and (B) intraneural and perineural increase of collagen-rich fibrotic tissue compared to (F,G) sham control and (K,L) scratch, for which only minor cell recruitment and loose perineural connective tissue were observed. Intraneural damage of the GG^high^ group was visible by (C) swelling of axons (arrows) and reduced amount thereof (circle) and (D) an overall decrease of Schwann cells as well as (E) demyelination. (M-O) In contrast, nerves treated with the cotton swab exhibited normal intraneural structure, similar to (H-J) sham control. Scale bars: 100 µm (high magnification) and 200 µm (inset).

In the GG^high^ group, Hematoxylin and eosin (H&E) staining exhibited strong cell infiltration combined with an increase of perineural connective tissue and a thickened epineurium (Fig. 3A, Fig. S3A), confirming the observed severe adhesions during re-exposure of the nerve. Also, intraneural fibrosis, identifiable as dense collagen-rich tissue, could be determined in this experimental group (Fig. 3B, Fig. S3B). These structural alterations could further be verified via immunostaining for neural markers. Anti-neurofilament protein staining demonstrated swollen or degenerated axons (Fig. 3C, Fig. S3C), as expected during Wallerian degeneration, including intraneural areas almost devoid of neurofilaments (Fig. 3C, black encircled). Beyond that, the amount of Schwann cells (Fig. 3D, Fig. S3D) and the degree of myelination (Fig. 3E, Fig. S3E) was clearly decreased, as shown with anti-S100 and Luxol fast blue staining, respectively. In contrast, the analysis of the scratch group revealed a widely normal histological structure (Fig. 3K-O, Fig. S3K-O). Merely an increase of mainly loose connective tissue surrounding the epineurium could be observed in some cases (Fig. 3L). Immunostaining for neural markers displayed a normal staining pattern similar to sham controls (Fig. 3H,I, Fig. S3H,I). As expected, histological analysis of the GG^low^ group revealed a locally restricted area of perineural fibrotic tissue as well as intraneural damage, which was less severe in comparison to the application of a larger quantity of glue, as in the GG^high^ group (data not shown).

### Sciatic nerve injury provokes immune-cell recruitment

**Fig. 4.**
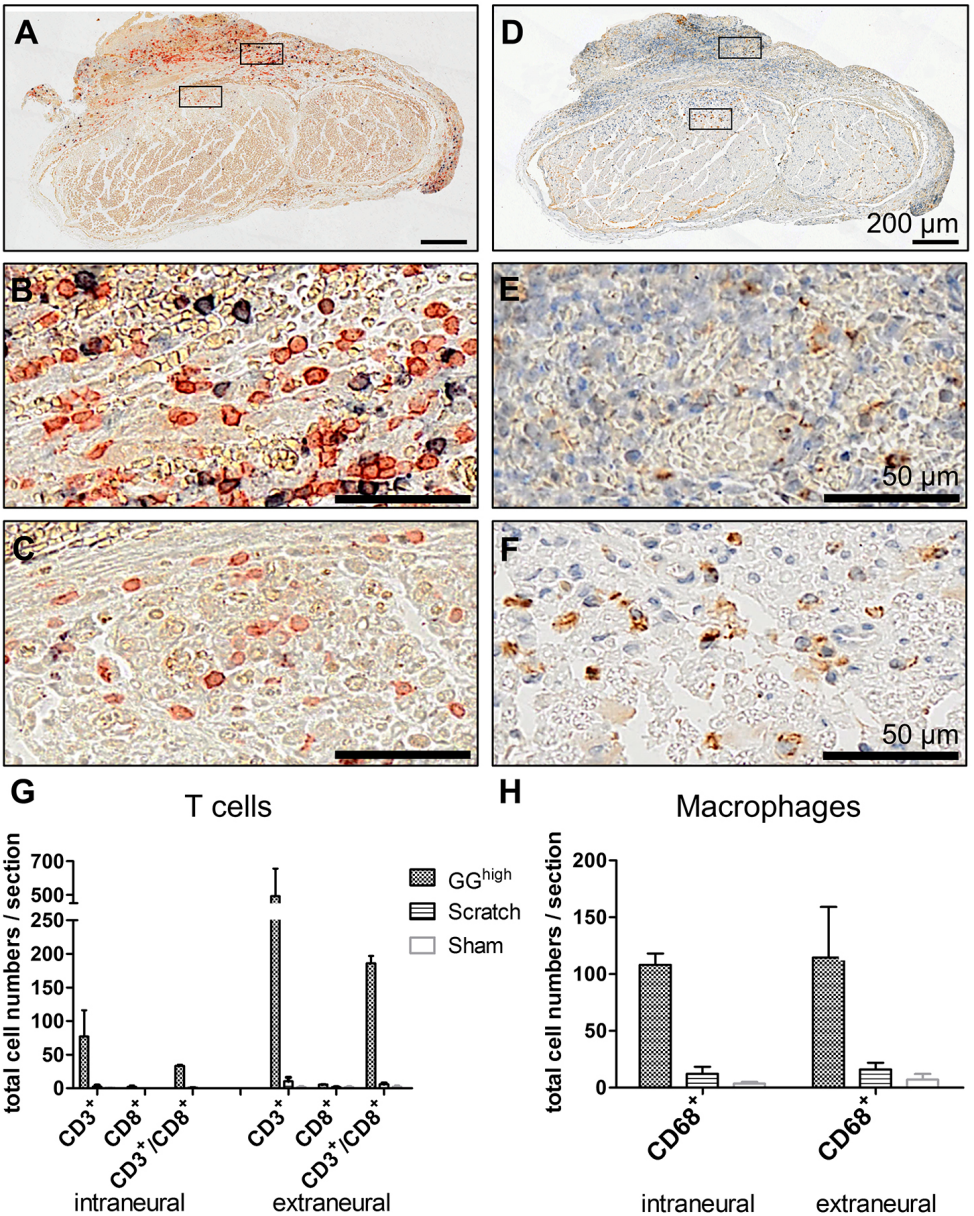
**T-cell and macrophage recruitment at the sciatic nerve 3 weeks after glutaraldehyde glue application.** (A-C) Cells were co-stained with antibodies against CD3 (red) and CD8 (blue) to detect T cells and (D-F) against CD68 (brown) for macrophages. Nuclei in D-F were counterstained with hematoxylin (blue). Quantification of cell numbers of representative cross sections revealed (G) high amounts of T cells and (H) macrophages inside and surrounding the sciatic nerve of glutaraldehyde-glue-treated rats in comparison to scratch and sham groups. Displayed are mean cell numbers with s.e.m. of respective histological sections of two rats of each experimental group.

In order to characterize the intense cell infiltration that was observed in histological sections, anti-CD3/CD8 co-staining (Fig. 4A-C) and anti-CD68 (Fig. 4D-F) staining was performed to visualize T cells and macrophages, respectively. As shown in Fig. 4, glutaraldehyde-containing glue induced a rigorous recruitment of these cells. T cells, of which a minor part were CD8^+^ cytotoxic T cells, invaded the epineurium, penetrated the blood–nerve barrier and could be seen between nerve fibres. Also, CD68^+^ macrophages were present around the epineurium and inside the nerve fascicle, mainly located between injured areas, to remove cell debris. In contrast, no or very few T cells and macrophages were found in sections of sham control and scratch animals (Fig. S4), which was further verified by quantification of these cells among the different experimental groups (Fig. 4G,H). This suggests that, owing to neural damage induced by glutaraldehyde, an innate and adaptive immune response was provoked, identified by strong T-cell and macrophage infiltration.

### Gait analysis reveals functional differences between groups

**Fig. 5.**
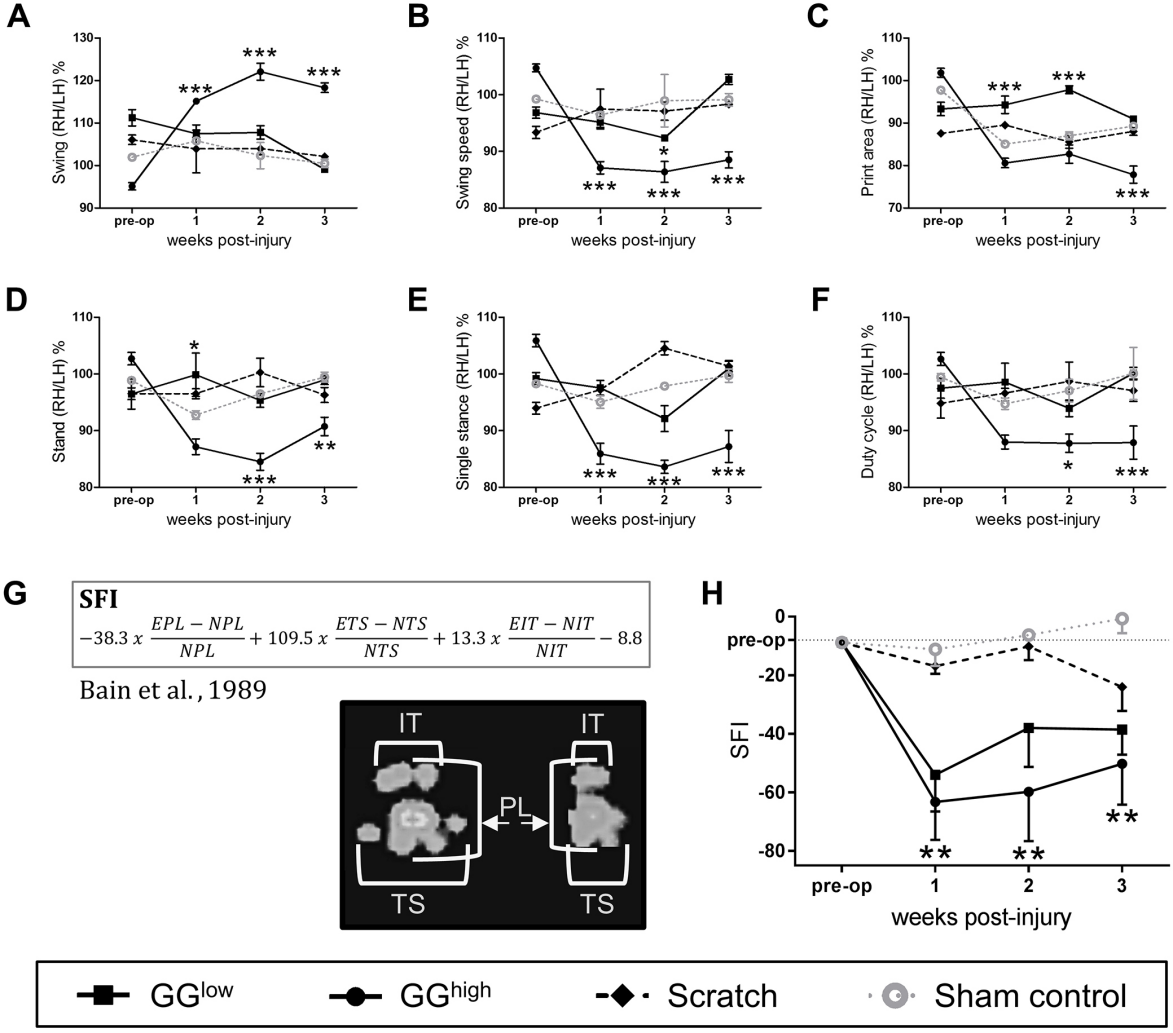
**Functional analysis of the sciatic nerve** **in** **the different experimental groups.** (A-F) Quantitative Catwalk™ gait analysis of motor function, including (A) swing, (B) swing speed, (C) print area, (D) stand, (E) single stance and (F) duty cycle over 3 weeks following primary surgery revealed gait impairment in the GG^high^ group compared to sham control, in contrast to rats of GG^low^ and scratch groups. *P<0.05, **P<0.01, ***P<0.001. Illustrated are the ratios as percentages between right (RH) and left hind limb (LH). *n*=6: scratch and sham control; *n*=8: GG^low^; *n*=11: GG^high^. (G) Formula for the calculation of the SFI according to Bain et al. (1989), and representative paw prints of the sham control (left) and GG^high^ group (right) 3 weeks after operation acquired with CatWalk™. E, experimental; N, normal; IT, intermediate toe spread; TS, toe spread; PL, print length. (H) Analysis of the SFI exhibited severe functional diminution of the sciatic nerve in the GG^high^ group compared to sham control starting 1 week after surgery. No severe functional deficiencies were detectable in the GG^low^ and scratch group; **P<0.01 GG^high^ compared with sham; *n*=6: scratch and sham control; *n*=8: GG^low^; *n*=11: GG^high^.

Diverse gait parameters, including swing, swing speed, print area, stand, single stance and duty cycle, were significantly affected in rats of the GG^high^ group compared with sham control (Fig. 5A-F). In most cases, these impairments were already obvious 1 week after glue application and persisted until the end of the observation period. Also, some parameters were altered within the GG^high^ drops group at early evaluation time points but had predominantly vanished by 3 weeks after primary surgery. This suggests that the application of a higher amount of glutaraldehyde glue induced a stronger impairment of the rat gait. Gait alterations within the scratch group did not exhibit any severe differences compared to sham control. To further characterize nerve function, the sciatic functional index was calculated according to Bain et al. (1989) by analyzing hind paw prints (Fig. 5G). As illustrated in Fig. 5H, in the GG^high^ group the sciatic nerve function [measured as sciatic functional index (SFI)] was already significantly impaired 1 week post-operation (−63.3±50.3) compared to sham control (−16.9±6.5, *P*=0.0037) and hardly improved until the final analysis. In the GG^low^ group, a decline of the sciatic function was also indicated; however, this was not statistically significant. The rats treated with the cotton swab displayed only minor functional alteration. These outcomes are summarized in Table 2.

**Table 2.**
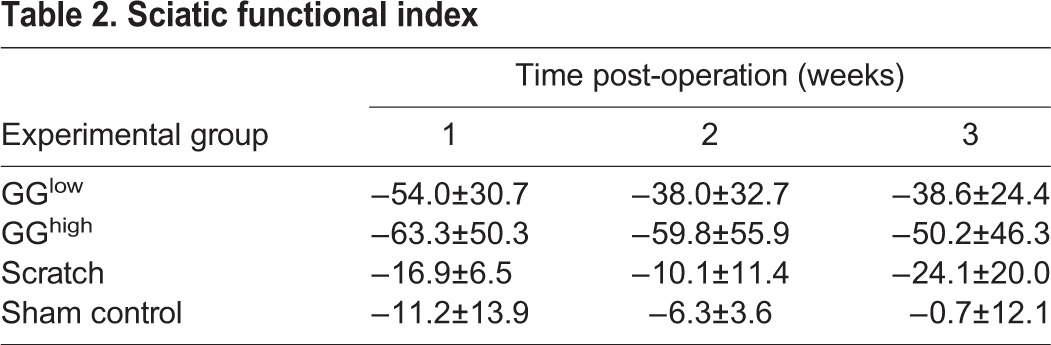
**Sciatic functional index**

### Severe secondary perineural adhesions occur after a defined amount of glutaraldehyde glue

**Fig. 6.**
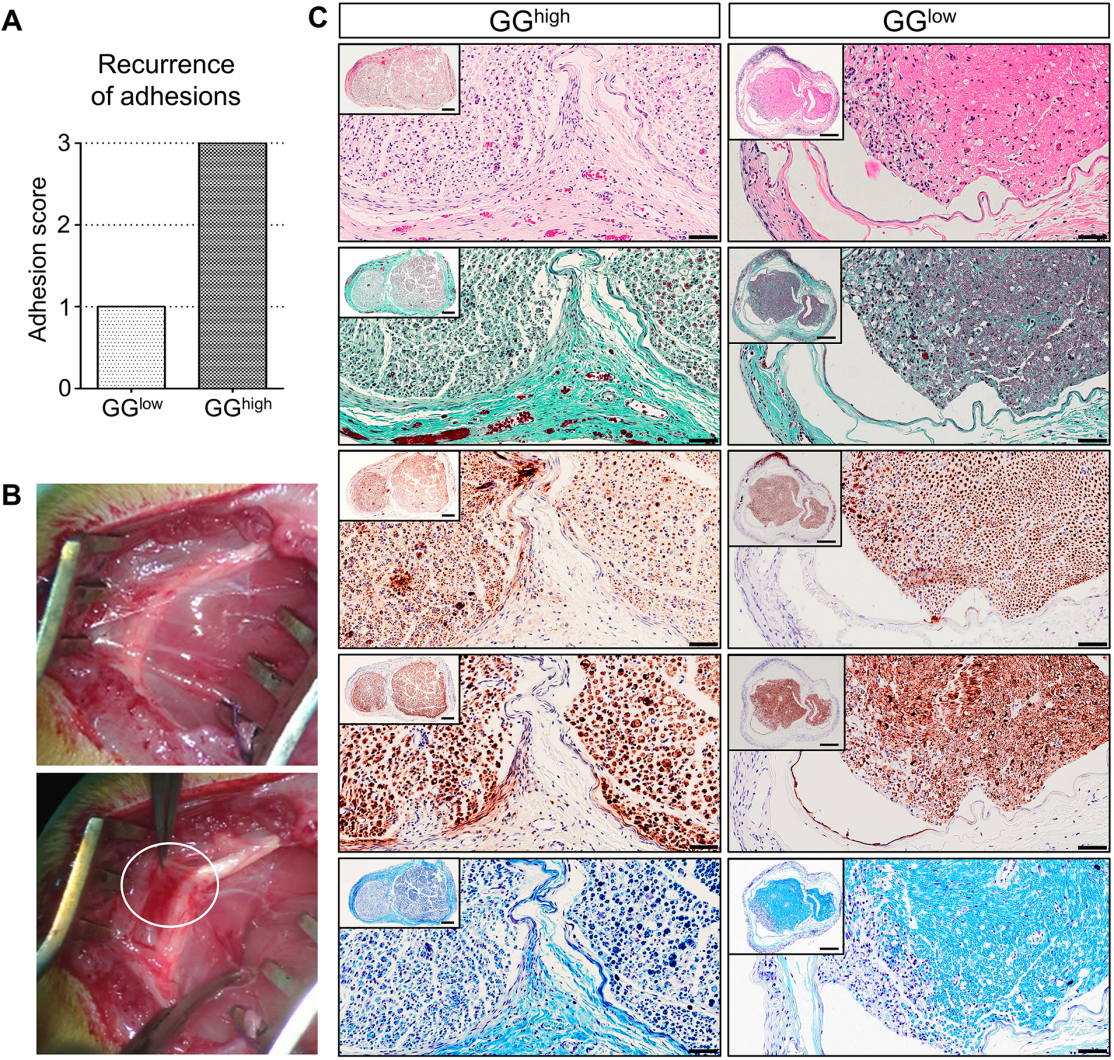
**Morphology of the sciatic nerve and secondary perineural adhesions 1 week after external neurolysis and glutaraldehyde glue removal.** (A) Comparison of recurrence of perineural scar tissue demonstrated severe adhesions within the GG^high^ group (right bar), in contrast to the GG^low^ group (left bar); *n*=2. (B) Gross morphological analysis revealed the formation of connective tissue (upper image) and regrowth of strong fibrotic fibers (lower image, encircled) 1 week after removal of 50 µl applied glutaraldehyde glue. (C) Representative cross sections of sciatic nerves of GG^high^ (left column) and GG^low^ (right column) were stained with H&E, Masson's trichrome, anti-neurofilament protein, anti-S100 and Luxol fast blue (from top to bottom). Comparison of both experimental groups revealed major differences in growth of perineural collagen-rich connective tissue and intraneural damage. Scale bars: 50 µm (high magnification) and 200 µm (inset).

Because the intention of this study was to establish a new and reliable rat model in which new therapeutic agents for the inhibition of secondary adhesions can be tested, the recurrence of perineural adhesions between sciatic nerve and surrounding tissue 1 week after external neurolysis and glue removal was analyzed. We observed that the application of glutaraldehyde glue drops was not enough to induce the re-appearance of distinct perineural fibrotic tissue until this time point, because blunt dissection was sufficient to remove the newly developed connective tissue (Fig. 6A). However, after applying at least 50 µl of glutaraldehyde glue, severe secondary adhesions, which needed sharp dissection, had already formed between the sciatic nerve and the surrounding tissue 1 week after glue removal (Fig. 6A,B). Confirming these findings, histological evaluation revealed not only a higher degree of intraneural degeneration but also increased fibrotic tissue formation in the GG^high^ group compared to the GG^low^ group (Fig. 6C). Hence, the application of a higher amount of glutaraldehyde-containing glue did not only induce greater intraneural damage, but also severe fibrotic perineural adhesions, recurring already one week after glue removal.

## DISCUSSION

Perineural scar formation and nerve fibrosis leading to impaired nerve function are major consequences of peripheral nerve injury. Also, perineural fibrotic adhesions, which are painful and hinder nerve regeneration, frequently reoccur (Atkins et al., 2006; Dam-Hieu et al., 2005; Frykman et al., 1981; Lundborg, 2003; Ngeow, 2010; Rydevik et al., 1976). Reliable animal models mimicking these complications, which cannot be explored via *in vitro* experiments, are still required. This makes it indispensable to have a robust and authentic model that simulates the most important clinical characteristics in order to assess the efficacy of urgently needed anti-adhesive and pro-regenerative therapeutic approaches for scarred peripheral nerves. The hitherto existing animal models either induce a direct or an indirect lesion to the nerve, such as irritating the nerve (Crosio et al., 2014; Görgülü et al., 2003) or burning the muscular bed (Abe et al., 2005; Albayrak et al., 2010; Crosio et al., 2014; Dam-Hieu et al., 2005; Finsterbush and Porat, 1982; Görgülü et al., 2003; Park et al., 2011; Smit et al., 2004; Tos et al., 2016; Zanjani et al., 2013; Zuijdendorp et al., 2008). The resulting pathological processes, mainly macroscopic and histological modifications, were described, but often no assessable functional impairment (Abe et al., 2005; Crosio et al., 2014; Dam-Hieu et al., 2005; Palatinsky et al., 1997; Smit et al., 2004; Tos et al., 2016; Yamamoto et al., 2010; Zanjani et al., 2013) – a significant clinical characteristic – was shown. One major difficulty might be the limited translatability of an animal model to human nerve degeneration. For instance, rats show a strongly increased regenerative capacity after nerve defects in comparison to humans (Derwin et al., 2010; Dobkin, 2007; Zanjani et al., 2013), which makes it challenging to create an adequate animal model. Nevertheless, the rat is still one of the most common species used for studies on nerve fibrosis (Zanjani et al., 2013).

One procedure in which distinct functional deficiencies can be observed in the rat is the transection of the sciatic nerve (Johnson et al., 2007; O'Neill et al., 2009; Özay et al., 2007; Özgenel, 2003). However, in this model the neural injury is extremely severe and functional recovery very slow. It does not, therefore, represent moderate intraneural degeneration and the resulting functional impairment in conjunction with perineural fibrotic adhesions that often result from surgical interventions in human patients. We compared our novel rat model with an already published procedure that induces perineural fibrotic adhesions by scratching the nerve surface with a cotton swab (Crosio et al., 2014). However, in our hands the scratch model did not lead to significant pathological conditions. Neither considerable histological alterations nor functional deficiencies were verifiable in any analyses we applied. The only alteration we could determine was the growth of loose connective tissue surrounding the nerve, which, however, required merely mild dissection. The difficulty in the reproducibility of the scratch model might be operator-dependency, because an objectively defined amount of pressure while applying the lesion is hardly possible, in contrast to our model in which a defined amount and concentration of the agent can be applied.

In our newly established rat model, we combined direct and indirect approaches for the chemical induction of lesions by applying a mixture of bovine serum albumin and glutaraldehyde, which consequently led to both intraneural fibrosis and perineural scarring. Two different application amounts were tested: (1) only one drop on either side of the nerve, to create a very locally restricted area of neural damage; (2) a larger but defined volume of glutaraldehyde glue (GG^high^) that covered 0.5-0.8 cm in length of the sciatic nerve and the surrounding muscles. This application induced the excessive *de novo* formation of connective tissue, encapsulating the glue, nerve and surrounding tissue. A 3-week contact period was necessary for the formation of severe adhesions, which required sharp dissection, although histopathological changes could be identified already 1 week after application. During external neurolysis after 3 weeks, the sciatic nerve appeared swollen, and Fontana bands were no longer visible. This is usually in itself a reliable indicator for intraneural or perineural fibrosis (Abe et al., 2005); indeed, (immuno-)histological analysis at different time points after application revealed peri- and intraneural fibrosis as well as clear indications for Wallerian degeneration, such as the breakdown of myelin and the disintegration of axons distal to the site of injury (Cajal and May, 1991; Fu and Gordon, 1997; Fawcett and Keynes, 1990; Namikawa et al., 2006), which were more intense after applying a larger amount of glutaraldehyde glue. The damaging part of this glue, glutaraldehyde, probably causes injury to the epi- and perineurium, consequently leading to the breakdown of the blood–nerve barrier. Immune cells, such as T cells and macrophages, were recruited, accumulated around the epineurium and entered the nerve, as is common during nerve degeneration (Beuche and Friede, 1984; Avellino et al., 1995; Cajal and May, 1991; DeFrancesco-Lisowitz et al., 2015; Gaudet et al., 2011; Dubový, 2011). Also, in the majority of the GG^low^ group, dense peri- and epineural scar tissue developed 3 weeks after application, as identified during re-exposure, although to a smaller extent than with a larger amount of the glue. Moreover, the intraneural damage was less pronounced in this group.

These histopathological indications were further reflected in electrophysiological and, very importantly, functional outcomes, which are relevant for translating the model into clinical significance (Deumens et al., 2007). The walking gait performance after application of the glue at higher amounts exhibited clear deficiencies in several gait pattern parameters, and these deficiencies began soon after application and persisted over the whole observation period. This was also reflected in the calculation of the SFI as according to Bain et al. (1989), another important parameter of functional performance, which was highly decreased in the GG^high^ group. By contrast, applying only two drops next to the nerve (GG^low^ group) was not sufficient to induce functional impairment. Similar to other animal models, the neural damage in the GG^low^ group, which was apparent via gross morphological and histological examination, could not be identified in a significant functional outcome. In the majority of publications, the analysis of functional alterations following perineural fibrosis is not described (Albayrak et al., 2010; Crosio et al., 2014; Ikeda et al., 2003; Ilbay et al., 2005; Ohsumi et al., 2005; Palatinsky et al., 1997; Park et al., 2011; Petersen et al., 1996; Smit et al., 2004; Tos et al., 2016; Yamamoto et al., 2010). Therefore, no conclusion can be made on whether the model is severe enough to induce functional deficiencies, which might be problematic for the evaluation of new therapeutic approaches because of the possibly insufficient clinical significance of the model. Moreover, histopathological changes frequently do not match with functional observations, as described in Zanjani et al. (2013). In their study, four different physical methods to induce perineural scar formation in the rat were tested. None of the interventions led to a significant impairment of the nerve function among the groups during the 4 weeks of follow-up, although perineural scar formation could be demonstrated histologically. This outcome reflects a major limitation of those animal models, because functional impairment is a key aspect of relevance for a model mimicking human perineural adhesions and nerve fibrosis. This demonstrates one main advantage of our new rat model: it was possible to reflect histomorphological and electrophysiological alterations in a functional examination.

In general, it was obvious that a larger amount of applied glutaraldehyde glue consequently led to a more pronounced course of disease. Not only can the location and defined amount of the applied glue be varied, but also the specific concentration of the harmful agent, glutaraldehyde, which was used at 2% in this study, can be modified. This concentration is commonly used in the commercially available surgical adhesive BioGlue, which is known to induce aortic growth impairment (LeMaire et al., 2002) and lung fibrosis (Haj-Yahia et al., 2007; Fürst and Banerjee, 2005), as well as nerve injury (LeMaire et al., 2007). An increased concentration of glutaraldehyde might lead to a stronger nerve lesion and more pronounced injury. Therefore, the possibility to vary volume and concentration of the glue makes this rat model also adaptable to specific needs, such as less- or more-severe neural injury.

Our intention was to generate a new model that induces not only primary perineural adhesions and fibrosis, but ideally should also lead to the spontaneous recurrence of scar tissue after external neurolysis, as often occurs in human patients with nerve injury. To our knowledge, no publication to date describes the reliable reformation of severe adhesions after removal of the connective tissue. Moreover, new treatments are often tested for the prevention of adhesions directly after inducing the damage (Albayrak et al., 2010; O'Neill et al., 2009; Okui et al., 2010; Özay et al., 2007; Özgenel, 2003; Park et al., 2011; Tos et al., 2016; Yamamoto et al., 2010). However, this does not represent the usual condition in human patients, who do not receive the anti-adhesion therapy directly after injury, but after formation or even after recurrence of adhesions. In our study we could indeed demonstrate severe secondary perineural adhesions 1 week after external neurolysis and the removal of a defined amount of 50-70 µl of glutaraldehyde-containing glue. Also, extensive histopathological alterations could still be identified at this time point, meaning that, in this model, new therapeutic approaches could be tested for regeneration after neural damage and to prevent the reformation of perineural adhesions.

### Conclusion

The application of glutaraldehyde-containing glue authentically induces intraneural inflammation, degeneration and fibrosis, as well as the formation of distinct perineural scar tissue, resulting not only in morphological and electrophysiological alterations, but also in quantifiable functional deficiencies. Moreover, this is the first model describing the recurrence of severe perineural fibrotic adhesions, thus inducing similar clinical conditions to those seen in patients. Taken together, our novel rat model reliably mimics all relevant pathological processes of peripheral nerve injury and therefore represents a very promising preclinical model for testing new therapeutic approaches for regeneration after nerve fibrosis and for the prevention of recurring perineural adhesions.

## MATERIALS AND METHODS

### Animals and experimental groups

All experimental protocols were approved in advance by the City Government of Vienna (Animal Use Permit No: MA58-358160/2015/17) in accordance with the Austrian law and the Guide for the Care and Use of Laboratory Animals as defined by the National Institutes of Health (revised 2011). A total of 38 female Sprague Dawley rats (Charles River, Sulzfeld, Germany), initially weighing 220-250 g, were randomly assigned into the following experimental groups: (1) glutaraldehyde glue (GG^high^, *n*=17), (2) GG^low^ (*n*=9), (3) scratch (*n*=6) and (4) sham-operated control (*n*=6) (see ‘Fibrosis-inducing procedure’ below for details of each group). The animals were provided with food and water *ad libitum*.

All animals were analyzed 3 weeks after primary surgery, except for the GG^high^ group, which had three different points of analysis: 1 (*n*=4), 2 (*n*=2) and 3 (*n*=9) weeks after surgery, to determine the time needed for adhesion formation by gross morphological evaluation.

To evaluate the formation of secondary adhesions, the sciatic nerves of four rats (GG^high^: *n*=2, GG^low^: *n*=2) were re-exposed 3 weeks after primary surgery and wounds closed after external neurolysis. These animals were analyzed after a further 1-week period.

### Surgical procedures

#### Fibrosis-inducing procedure

All animals were anesthetized using isoflurane (Forane^®^; AbbVie Ltd, Berkshire, UK). The right hind limb was shaved and a 2- to 3-cm long incision set on the right lateral thigh. Under aseptic conditions, the right sciatic nerve was carefully exposed at midthigh by way of a lateral approach and isolated from the surrounding tissue. In the GG^high^ group, a mixture of 35% bovine serum albumin (Sigma Aldrich, St Louis, MO, USA) and 2% glutaraldehyde (AppliChem, Maryland Heights, MO, USA) (‘glutaraldehyde glue’) was applied on top of the sciatic nerve as well as the surrounding muscles at a dosage of 50-70 µl over a length of 0.5-0.8 cm. In the GG^low^ group, only two drops of 5-10 µl each of the glutaraldehyde-containing glue were administered, one ventral and one dorsal to the nerve, to ensure the local restriction of neural damage. As a comparison, an already published procedure for fibrotic adhesions was performed, similar to Crosio et al. (2014). The sciatic nerve was scratched 20× with a sterile cotton swab (Noba, Wetter, Germany) over a length of 0.8 cm to induce fibrosis (scratch group). As a control, sham operations were conducted in which the sciatic nerve was exposed and isolated from the surrounding tissue only. Afterwards, the wound was closed and the skin sutured using absorbable suture material (4-0 Vicryl^®^; Johnson & Johnson Medical GmbH, Norderstedt, Germany). All surgical procedures were performed under an operating microscope (Leica M651; Leica Microsystems, Vienna, Austria) and a heating plate was used to ensure the maintenance of body temperature. Meloxicam (0.2 mg/kg body weight Metacam^®^, Boehringer Ingelheim Vetmedica, Inc., St Joseph, MO, USA) was given as a preemptive and postoperative analgesic for 4 days. Buprenorphine (0.05 mg/kg body weight Bupaq, Richter Pharma AG, Wels, Austria) was applied as a postoperative analgesia. After recovery from anesthesia, the animals had access to water and food *ad libitum*.

#### External neurolysis during re-exposure of the sciatic nerve

A skin incision and transection of the femoral fascia as performed during the fibrosis-inducing operation was conducted. Connective tissue between the muscle and sciatic nerve, which developed after the first surgery, was then removed and the nerve re-exposed to enable electrophysiological analysis. Moreover, the glutaraldehyde glue of the GG^high^ and GG^low^ groups was removed. During this procedure, the degree of perineural adhesions was graded. The animals that were assigned to en bloc histology did not undergo external neurolysis.

### Gross evaluation of adhesions

At the time point of analysis, adhesions were evaluated during re-exposure of the right sciatic nerve using a numerical grading scheme as defined by Petersen et al. (1996). The grading was performed by the surgeons (C.P. and J.F.), who were blinded to the experimental groups. Perineural adhesions with the surrounding muscles and nerve separability of the respective rat were classified into three different categories: grade 1 reflected no or only mild adherence, grade 2 indicated the necessity for stronger but still blunt dissection and animals assigned to grade 3 required sharp dissection to separate the sciatic nerve from the surrounding tissue. Sciatic nerves that were used for en bloc histology were not re-exposed and therefore not graded.

### Histology and immunohistochemical analysis

Two different procedures were performed. For en bloc analysis of the sciatic nerve and perineural adhesions to the surrounding tissue, rats in deep anesthesia (*n*=4) were perfused with a fixative solution as published (Gage et al., 2012). Afterwards, nerve and surrounding tissue were carefully removed en bloc and immersed for another 48 h in the fixative before processing for histology. In all other rats, the exposed nerve was carefully dissected and immersion-fixed in 4% buffered formaldehyde (VWR, Radnor, PA, USA) overnight after euthanization. After washing for at least 1 h in water, the nerves (dissected and en bloc) were transferred first into 50% ethanol, then into 70% ethanol. Then, the specimens were embedded in paraffin, 4-µm-thick sections sliced with a rotary microtome, dried at 40°C overnight, deparaffinized in xylene and rehydrated in a graded series of alcohol. For histological analysis, sections were stained with hematoxylin and eosin (H&E), Masson's trichrome, which stains collagen green, as well as Luxol fast blue, which labels myelin blue. En bloc cross sections were stained with chromotrop-aniline-blue (CAB), visualizing collagen in blue and muscle fibres in red.

Axons and Schwann cells were detected by applying mouse monoclonal anti-human neurofilament protein clone 2F11 (1:100) and polyclonal rabbit anti-S100 (Z0311, 1:1600) (both from DAKO, Glostrup, Denmark), respectively. Immune cells were stained using mouse monoclonal anti-CD68 (macrophages; MCA341R, 1:10,000, Serotec, Puchheim, Germany), rabbit polyclonal anti-CD3 (T cells; RM-9107-S, 1:100, NeoMarkers, Fremont, CA, USA) and mouse monoclonal anti-CD8 (cytotoxic T cells; MAS041, 1:200, Serotec, Puchheim, Germany).

The number of CD68^+^, CD3^+^ and/or CD8^+^ cells was quantified using ImageJ.

### Gait analysis (CatWalk™ XT)

To detect possible functional deficiencies after fibrosis induction, gait analysis was performed using CatWalk™ XT (V10.6, Noldus Information Technology, Wageningen, The Netherlands). The use of this software enables the objective examination and quantification of various static and dynamic parameters concerning the gait pattern of the rat (Deumens et al., 2007). The animals were trained prior to the primary surgery in order to grow accustomed to the device. After that, at least three evenly performed and consistent runs were taken as baseline. Postoperatively, the gait was assessed once a week until the end of the survival period. To identify gait alterations among the experimental groups, the results of the right hind limb were expressed as a ratio of the left hind limb, and the mean of at least three compliant runs comprising three representative prints of every paw were taken.

### Quantification of the sciatic functional index (SFI)

In addition to the analysis of the rat gait, the function of the sciatic nerve was quantified using the calculation of Bain et al. (1989). For this, toe spread, intermediate toe spread and print length of the right hind paw of at least three runs were determined using CatWalk™ XT and the mean taken, identifying an impairment of the sciatic nerve function as a decrease in the index value. As a control print (‘normal’), the mean values of the footprints recorded during the baseline trials of the respective rat were used, which resulted in an SFI of –8 pre-surgically.

### Electrophysiology

At the end of the survival period, electrophysiological analysis (NeuroMax-XLTEK, Oakville, Ontario, Canada) was performed under anesthesia prior to euthanization, by placing stimulation electrodes proximal and distal to the nerve lesion. One electrode was positioned in the tibialis anterior muscle as recording electrode and the supramaximal stimulation amplitude was verified by stimulation for 0.05 ms. Peak amplitude and NCAP were determined. To reduce possible anesthesia effects, data were presented as the ratio of the right to left hind limb values.

### Statistical analysis

Statistical analysis was performed using GraphPad Prism 7.0 (GraphPad Software Inc., San Diego, CA, USA). The data was tested for normal distribution using the Kolmogorov–Smirnov test. Normally distributed data was statistically analyzed using either Student's *t*-test (two groups to compare) or one-way ANOVA and Bonferroni's multiple comparison *post hoc* test. Otherwise, the Kruskal–Wallis test followed by Dunn's multiple comparison *post hoc* test was applied. To compare various experimental groups among different time points, two-way ANOVA and Bonferroni's multiple comparison *post hoc* tests were used. Results are expressed as means with standard error mean (s.e.m.) and a *P*-value of <0.05 was considered as statistically significant.
